# Growth and characteristics of self-assembled MoS_2_/Mo-S-C nanoperiod multilayers for enhanced tribological performance

**DOI:** 10.1038/srep25378

**Published:** 2016-05-03

**Authors:** Jiao Xu, TengFei He, LiQiang Chai, Li Qiao, Peng Wang, WeiMin Liu

**Affiliations:** 1 State Key Laboratory of Solid Lubrication, Lanzhou Institute of Chemical Physics, Chinese Academy of Sciences, Lanzhou 730000, People’s Republic of China

## Abstract

Highly ordered MoS_2_/Mo-S-C nanoperiod multilayers are synthesized by a novel self-assembling mechanism in simultaneous sputtering of MoS_2_ and graphite targets. The sequential formation of MoS_2_-riched domain layers and Mo-S-C compositional mixed capping layers reveals no correspondence to the sample stage rotation but is caused by the low energy ion bombardment enhanced interdiffusion. The HRTEM observation shows that the phase segregation normal to the film surface is initiated from substrate-film interlayer with clear contrasts in the first few bi-layers, and then diffuses mutually in a quasiperiodic pattern between two altered sub-layers. Compared with sputtered MoS_2_ film, the bulk film of multilayers exhibit largely improved toughness under a normal load, and the preferential orientation of sputtered MoS_2_ in (002) basal planes is significantly enhanced, both of which render the film excellent loads-bearing capacity and lubricant properties. The nano-scratching tests performed on a nanoindentation system suggest that the nano-tribological performance of multilayers is directly determined by the altered structure and properties of neighboring sub-layers until stable tribofilms are formed. Meanwhile, the pin-on-disk tribotests in ambient air, low vacuum and high vacuum provide comparably low friction coefficient yet distinct wear lives in different atmospheres due to the partially restricted humid-sensitivity of sputtered MoS_2_ phase.

Nanostructured film has attracted much attention in recent years due to its extraordinary properties and potential applications in functional materials and nanodevices. Of particular interest is to fabricate the superlatticed films at nanometer scale through self-assembling processes during the thin film deposition^1^. Typically, the spontaneous formation of superlattice structures have been reported in Si_1−x_Ge_x_ films prepared by molecular beam epitaxy (MBE) assign to the modulated local strain field^1^, the Au-Ni alloys prepared by ion bean assisted deposition (IBAD) assign to the supersaturated mutual solubility and spinodal decompostion^2^, the III-V and II-VI alloys prepared by MBE^3^,^4^,^5^ and the Si_1−x_C_x_ films prepared by gas-precursor MBE due to the self-arresting feedback loop in surface-reaction pathways^6^. However, the atomic mechanism of self-assembling process is still under debate.

Recently, self-assembled superlattice structures have been observed in metal-doped amorphous carbon films^7^,^8^,^9^,^10^,^11^,^12^,^13^,^14^ in simultaneous co-sputtering of both species and triggered great interest in the field of functional thin films. Wu and Ting *et al*.^7^,^8^,^9^ prepared one set of self-assembled multilayers of a-C:Me (Me including Al, Si, Fe, Ni, Cu, Pt, Au) by dc reactive magnetron sputtering and mass selective ion beam deposition (MSIBD) through appropriate controlling of impinging ion energy *E* and ion ratio *r* of carbon and metal ions. Their HRTEM observations suggested in particular that the carbide formation ability of doped metal plays an important role in spontaneous formation of multilayers. The Al, Si with strong solid solubility to carbons formed uniform amorphous carbide films, while the weak carbide former Fe and Ni exhibited significant altered layer structure of metal-rich and metal-deficient layers, and the non-carbide former Cu, Pt and Au showed the clearest layer sequence of metal-rich and metal-deficient sheets initiated from substrates. Similar conclusions were withdrawn by Gerhards and Ronning *et al*.^10^,^11^ in their investigation on self-assembled a-C:Me (Me including Cu, Ag, Au) multilayers deposited by the same methods. By comparison, Hosson and his partners^12^,^13^ achieved tunable growth of self-organized TiC/a-C nanocomposite multilayers by nonreactive dc magnetron sputtering of Ti and graphite targets on biased Si substrates. They found that^12^ the phase separation of TiC nanocrystallites and a-C capping layers was determined by a complex interplay among a) the subplantation through enhanced diffusion under intensified ion impingement, b) the surface roughness of sub-layers and c) the random nucleation on growing front layer. Similarly, Hovsepian *et al*.^14^,^15^,^16^ deposited self-organized CrC/a-C multilayers by combined steered cathodic arc/unbalanced magnetron sputtering of Cr and graphite targets. It seems that the self-assembled compositional modulation of transition metal/a-C films are mostly formed as metal-carbide nanocrystallites and a-C altered multilayers.

Presentatively analogous to the abovementioned cases, a self-assembled multilayer structure with 1~2 nm sub-layer thickness had been observed in r.f. co-sputtered WS_2_/a-C composite films as once reported by Noshiro *et al*.^17^ Well-laminated nanostructures with 1~2 nm sub-layer thickness were obtained by r.f. co-sputtering of WS_2_ and graphite targets, and the film showed weakened hardness but lower friction in ambient air as compared with pure a-C films^17^,^18^,^19^. A similar nanostructure was seen in MoS_2_/a-C composite films as glanced in ref. 17, 18, 19, however, without clear descriptions and discussions on its influence on the characteristics of multilayered films. In fact, compared with the self-assembled Me/a-C^7^,^8^,^9^,^10^,^11^ multilayers and transitional metal-carbide/a-C multilayers^12^,^13^,^14^,^15^,^16^ generally prepared by high energy ion impingement, more careful considerations are acquired in investigation on self-assembled WS_2_/MoS_2_/a-C multilayers, since in this case relatively lower impinging energy is applied, so that the intrinsic lamellar structure of WS_2_/MoS_2_ phases can be preserved to ensure the low friction performance of deposited films. In principle, the self-assembling process occurring in WS_2_/MoS_2_/a-C multilayers is primarily caused by the low energy ion bombardment enhanced phase segregation.

In this work, we for the first time to our knowledge, report self-assembled MoS_2_/Mo-S-C multilayers with distinct nanoperiodicities of MoS_2_-riched domain layers (in 8~9 nm thick) and Mo-S-C compositional mixed capping layers (in 2~4 nm thick) by r.f. co-sputtering of MoS_2_ and graphite targets onto non-biased substrates. Cross sections of the films were observed by using high resolution transmission electron microscopy (HRTEM), and the composition and structure of the films were investigated by using X-ray diffraction spectroscopy (XRD) and Raman spectroscopy. Taking account of the laminated structure normal to the film surface at nanoscale, the nano-tribological property of deposited film was checked by using a nano-scratching tester, while a pin-on-disk tribometer was also applied for comparison. In particular, the transferred materials derived from pin-on-disk tribotests were analyzed by HRTEM, which surprisingly showed finely-ordered MoS_2_ molecular layers stacked approximately in 30 nm thick in parallel to sliding direction, acting as durable protective layers between sliding counterfaces.

## Results and Discussions

### Morphology and Structure characterizations

**Figure 1:**
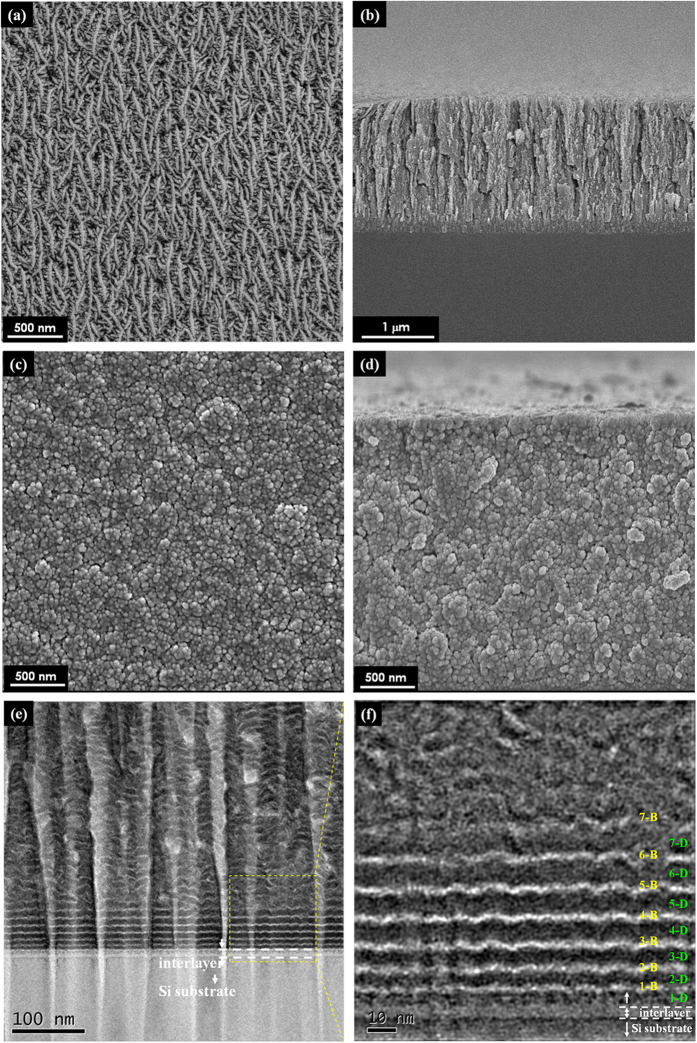
The FESEM and HRTEM observations of referred pure sputtered MoS_2_ film and MoS_2_/Mo-S-C multilayers. (**a,b**) the FESEM images of top surface and fractured cross-section of pure sputtered MoS_2_ film with a pre-sputtered Ti interlayer in thickness of 200 nm; (**c,d**) the FESEM images of top surface and fractured cross-section of nanoperiod multilayers.; (**e,f**) the cross-sectional HRTEM observations of nanoperiod multilayers with gradual increasing sub-layer thickness of the first seven bi-layers above the film-substrate interlayer.

The FESEM images of top surface and fractured cross-section of referred pure sputtered MoS_2_ film and MoS_2_/Mo-S-C multilayers are shown in Fig. 1(a–d). Compared with MoS_2_ film usually exhibiting porous and columnar structure^20^,^21^, the multilayered film exhibits denser structure composed of stacked particles in microscale, and the cracks along the direction of film growth are significantly blocked. Further observations performed by HRTEM in Fig. 1(e) reveal that the film exhibits a nanoperiod multilayer structure initiating from the film-substrate interface with equally well-defined contrast for tens of nanometers, and then gradually diming due to the large surface roughness of sub-layers and the random nucleation on growing front surface^12^,^13^. Nonetheless, typical characteristics of multilayer structure can be recognized throughout the entire thickness of the film, which show distinct nanopriorities of the domain layer (in dark, denoted as -D in Fig. 1(f) and later) and the capping layer (in bright, denoted as -B) altered in waving patterns. Closer inspections of the initiating region above film-substrate interlayer confirm a gradually increasing thickness of the first seven bi-layers, approximately from 4 nm to 9 nm for the dark sub-layers and from 2 nm to 4 nm for the bright sub-layers as listed in Table 1; and afterwards, the sub-layer thickness becomes saturated and keeps stable.

### Structure and crystallography characterizations of multilayers on nanoscale

**Figure 2:**
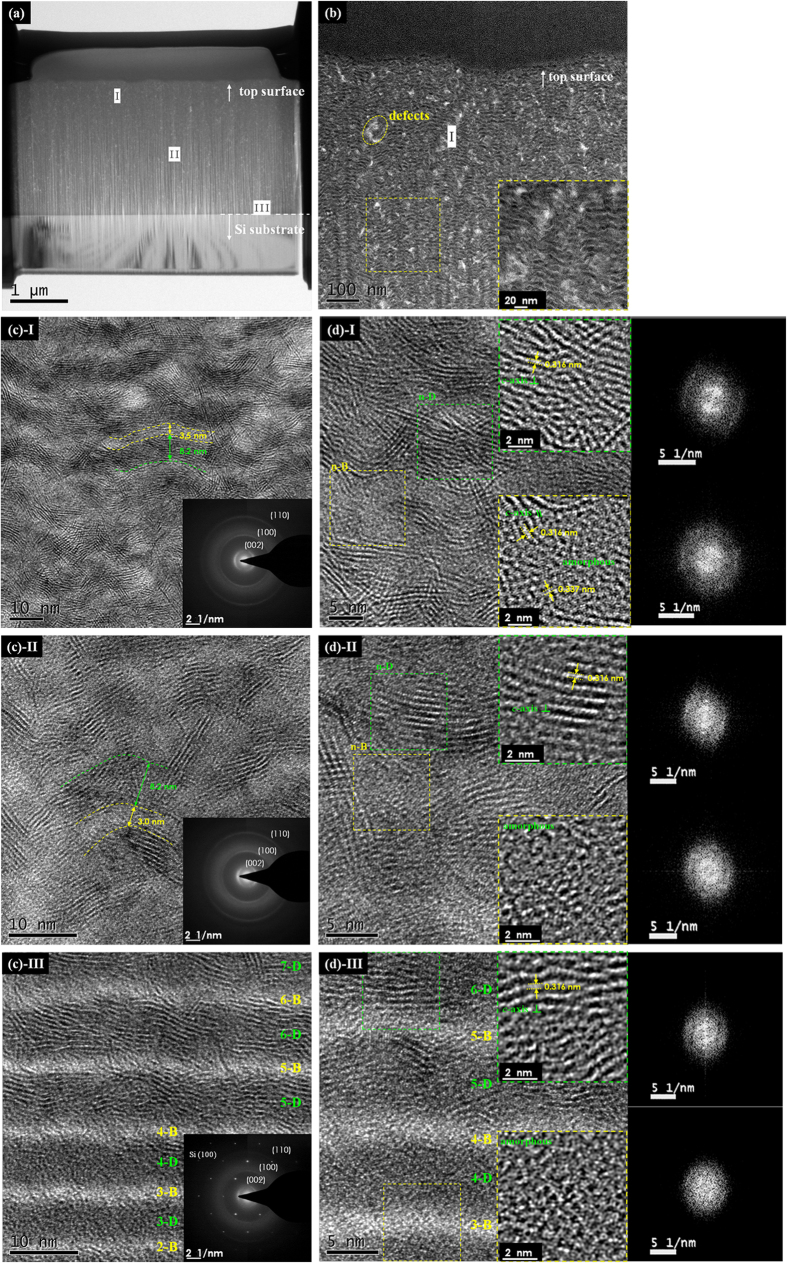
The cross-sectional HRTEM observations and closer inspections of nanoperiod multilayer structure in regions I–III along the direction of film growth. (**a**) The FIB-prepared slice for HRTEM experiment and the selected regions for comparison. (**b**) The overview graph of region I, with inset showing the high-magnification image. (**c**)**-I**, (**c**)-**II** and (**c**)-**III**, the multilayer structure in region I, II and III, respectively, with the insets showing the corresponding SAED patterns. (**d**)**-I**, (**d**)**-II**, (**d**)-**III**, the high-magnification images of region I, II and III, respectively; the high and low insets show the inverse filtered fast Fourier transformation of selected parts in dashed frames, and the corresponding fast Fourier transformation representing the local diffraction patterns are added at the right side.

Carefully, in Fig. 2(a) three regions representing the film top surface, in-between region and film-substrate interface, coded as I, II and III respectively, were selected along the film thickness direction and intensively observed. The overview graph of region I shown in Fig. 2(b) confirms the multilayer structure throughout the entire thickness of the film with saturated sub-layer thickness as mentioned above. The defects (the bright spots in Fig. 2(b)) mostly structured in localized amorphous phase are embedded in-between 1–4 bi-layers depending on their dimensions. Further inspections of the three regions, as demonstrated in Fig. 2(c,d) I–III, suggest an ordering tendency of the domain layers from amorphous to cross-linked molecular layers with the film growing, particularly within the first five bi-layers as seen in Fig. 2(c)-III,(d)-III. Consistently, the SAED pattern of region I (inset of Fig. 2(c)-I) shows clear diffraction rings of sputtered MoS_2_ phase dominant in (002) orientation, together with the weak rings of (100) and (110) orientations and the diffraction halo possibly assigned to the a-C phase. Closer observations of region I, highlighted by inverse filtered fast Fourier transformation as shown in Fig. 2(d)-I and its insets, reveal that the domain layers in thickness about 8 nm are mainly composed of cross-linked MoS_2_ molecular layers (monolayer thickness of 0.316 nm) roughly in parallel to the film surface in a waving pattern, while the capping layer in thickness about 3 nm are composed of highly distorted MoS_2_ clusters, visible graphitic fragments (monolayer thickness of 0.337 nm) and amorphous carbons in a mixed manner. Also, the filtered fast Fourier transformation of dash-squared parts of region I, which represents the local diffraction pattern as given in the right side of Fig. 2(d)-I, confirms again the preferred (002) orientation in MoS_2_-riched domain layers and the weakened crystallinity in compositional mixed capping layers. Compared with region I, few changes are waked in region II with regard to the reduced crystallinity both in the domain and capping layers, as reflected by the insets and diffraction patterns in Fig. 2(c)-II,(d)-II. By contrast, obvious transformations of the layer structure and crystallography occurred in region III, i.e. the initiating stage of self-assembling process. In Fig. 2(c,d)-III, except for the gradient increase of bi-layer thickness, the structure of domain layers steps from amorphous to partial clustering above the fourth bright layer 4-B, and then tends to be cross-linked MoS_2_ molecular layers in well alignment.

**Figure 3:**
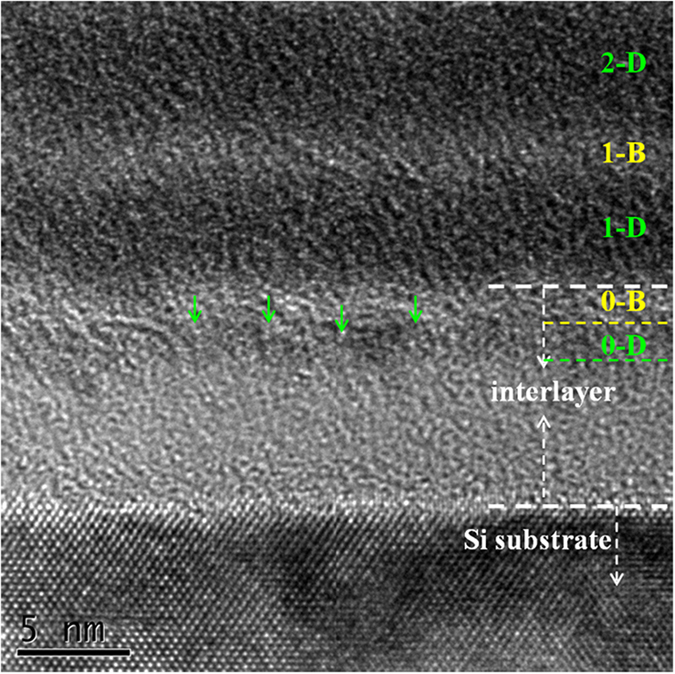
The cross-sectional HRTEM observation of the self- assembled interlayer between the deposited film and Si substrate.

Concerning about the self-assembling mechanism of MoS_2_/Mo-S-C multilayers derived from low energy ion bombardment, a close-up profile of the interlayer is captured as given in Fig. 3. Noticeably, a discontinuous penetration of the 1-D layer can be observed at the frontier of the interlayer (noted by arrows) and thus forms two mutual-diffused stripelike layers in thickness of 2 nm, which are tentatively denoted as 0-B and 0-D, respectively. According to the previous work by A.H. Eltoukhy and J.E. Greene^22^,^23^, the depth dependent interdiffusion coefficient D^*^(x) can be enhanced even at non-biased sputtering condition due to the substrate potential of positive space-charge region in Ar discharge, and as a result, compositional altered layers may be formed and initiated at heterojunction interfaces. Next in important, enhanced diffusion has a significant effect on the altered layer thickness and the total ion dose acquired to reach steady-state multilayer structure during ion etching of the growing surface. Hereby, in previous reports^12^,^22^,^23^ as well as the present work, the thickness of altered layers shows weak relations with the passing time of substrates through sputtered targets, but can be effectively tuned by the sputtering power and substrate bias voltage^12^. Furthermore, as the diffusion coefficient D*(x) can be enhanced by large concentrations of point defects, i.e. vacancies, interstitials and boundaries of nucleation which are particularly rich in film-substrate interlayer region III, the thickness of bi-layers in the initial stage is relatively smaller and getting larger within the first seven bi-layers until a steady state is reached^12^,^23^.

### Crystallography, composition and mechanical property characterizations of bulk film

**Figure 4:**
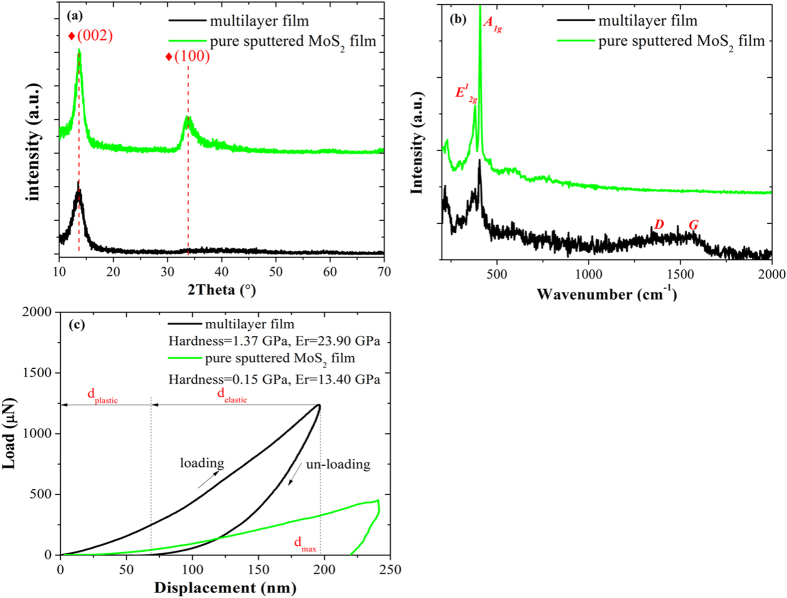
(**a**) The GIXAD pattern, (**b**) the Raman spectra and (**c**) the nanoindented load-displacement curves of the bulk film.

The crystallography structure of bulk film was characterized by using GIXRD and the results are shown in Fig. 4(a). Compared with pure sputtered MoS_2_ film with Bragg (002) peak at 13° and (100) peak at 33*°*, the (002) peak of composite film becomes broadened due to the loss of long-range ordering of MoS_2_ crystallites, and the (100) peak is restricted exhaustively. Such a tendency had also been observed in our previous study of MoS_2_/a-C composite films with various contents of carbon^20^. In principle, the overwhelming preference of (002) orientation in parallel to film surface may enhance the lubricant property of bulk film in vacuum and dry atmospheres^24^,^25^.

Figure 4(b) shows the composition characterization of bulk film by Raman spectroscopy. Two modes of first-order Raman activity of MoS_2_ component induce well-defined bands of *E*^*1*^_*2g*_ and *A*_*1g*_ centered at 380 cm^−1^ and 408 cm^−1^, respectively. The *E*^*1*^_*2g*_ band is attributed to the motion of Mo+ S atoms in the *x−y* layered plane, and the *A*_*1g*_band is attributed to the motion of S atoms along the *z* axis^26^. Compared with pure sputtered MoS_2_ film, the two bands of composite film get broadened and the two bands shift to 371 cm^−1^ and 404 cm^−1^, respectively, primarily attributed to the weakened crystallinity and bond distortion in MoS_2_ crystallites^20^,^26^,^27^,^28^. Meanwhile, in the spectrum of composite film, the *D* and *G* peaks of carbon phase are seen at 1390 cm^−1^ and 1567 cm^−1^, respectively, suggesting an amorphous structure of incorporated carbons in composite film^29^.

In addition, the mechanical property of bulk film is demonstrated in Fig. 4(c). Compared with pure sputtered MoS_2_ film, the hardness of composite film was improved by one order of magnitude higher, and the elastic recovery ratio *d*_*elastic*_*/d*_*max*_ increased significantly to 67%. However, reviews on previous work of MoS_2_/a-C composite films^20^,^30^,^31^,^32^,^33^ suggest that such an increase of film hardness may dominantly assigned to the densification of film structure in microscale, so that the hardening effect induced by multilayer structure cannot be assured in this work.

### Nano-tribological property characterizations

**Figure 5:**
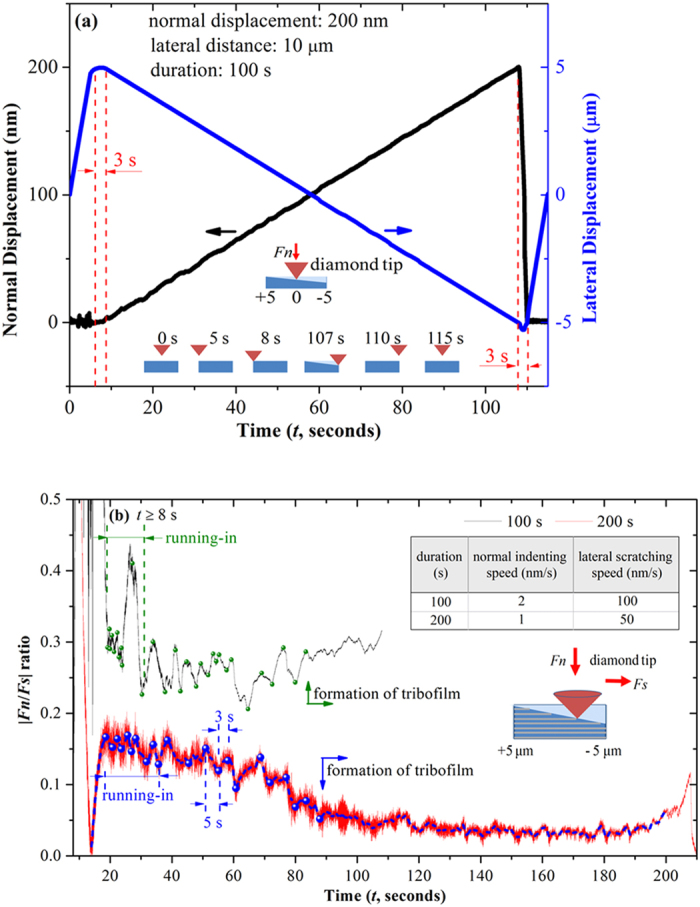
The nanoscale scratching tests of nanoperiod multilayers performed on a standard nanoindentation system. (**a**) The developments of normal and lateral displacements as functions of time when the scratching duration was set as 100 s, and (**b**) the |*F*_*n*_*/F*_*s*_| ratio curves of nanoscratching tests (*t* ≧ 8s) as the scratching durations were set as 100 s and 200 s.

To classify the influence of multilayer structure on the nano-tribological property of deposited film, a standard nanoindentation system was applied in a gradual-depth-increase scratching mode in ambient air atmosphere at room temperature. The indentation durations were set as 100 s and 200 s for two contrastive studies, so that the indentation speeds of diamond tip were 2 nm/s and 1 nm/s, respectively. Taking 100 s test for example, developments of the normal and lateral displacements of the tip are shown in Fig. 5(a) as functions of time. The tip position was initiated laterally at zero (i.e. at the center) and normally hanged above the scratched surface. In the next, the tip moved horizontally to +5 μm in 5 s, taking 3 s to approach the tested surface, and then started the scratching test from +5 μm to −5 μm in lateral direction; simultaneously, the tip indented from the top surface to 200 nm in normal direction. Conversely, as the scratching was finished, the tip detached from the film in 3 seconds and then moved horizontally back to the initial position in the next 5 s.

In Fig. 5(b), the curves of |*F*_*n*_*/F*_*s*_| ratio were recorded as 8s ≦*t* ≦107 s (8s ≦*t* ≦199 s for 200s test, *F*_*n*_ the normal load, *F*_*s*_ the shearing force), which represent the developments of friction coefficient with the periodic contacts between the tip and domain layers or capping layers in increasing depth. Interestingly, as might be expected, a quasiperiodic waving of the ratio could be seen in both of the two cases after a running-in period approximately for 15s. In case of 100s test, both the lateral scratching speed and the normal indenting speed were relatively higher, so that the contacts between the tip and scratched surface were more susceptible to the altered structures and mechanical strengths of neighboring sub-layers. As a result, large fluctuations are seen in the curve of |*F*_*n*_*/F*_*s*_| ratio with higher average value about 0.25, and the formation of lubricant tribofilm takes longer scratching length about 8 μm. By comparison, in 200s test the fluctuations of |*F*_*n*_*/F*_*s*_| ratio are obviously moderate with up and down quasi-periodicities as 3 s and 5 s, respectively, which are roughly consistent to the saturated thickness of compositional mixed capping layers and MoS_2_-riched domain layers. Meanwhile, the formation of lubricant tribofilm is clearly indicated by the stable tendency of |*F*_*n*_*/F*_*s*_| ratio after 90 s (i.e. 450 nm in length) scratching. Hereby, it can be deduced that the nano-tribological performance of multilayered film is directly determined by the altered structure and properties of neighboring sub-layers until stable tribofilms are formed on sliding counterparts.

### Tribological property and lubricant mechanism of bulk film

**Figure 6:**
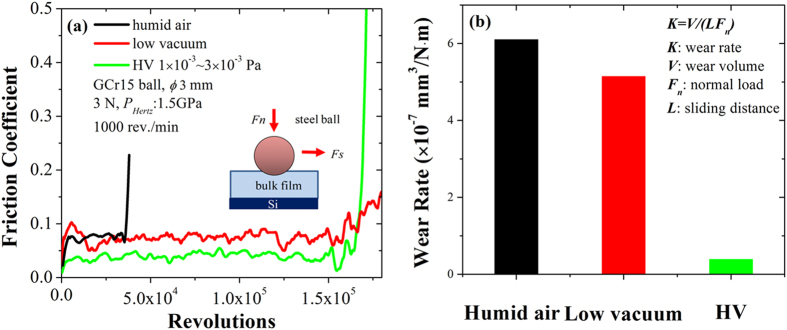
The pin-on-disk sliding tests of deposited film. (**a**) The friction coefficient curves in humid air, low vacuum and high vacuum, and (**b**) the film wear rates evaluated by performing tribotests for 2 × 10^4^ revolutions.

The pin-on-disk tribology tests were performed in humid air, low vacuum and high vacuum successively under the same loading conditions. The theoretical Hertz contact stress *P*_*Hertz*_ is 1.5 GPa, which is comparable to the mechanical strength of bulk film. Figure 6(a) depicts the friction coefficient curves derived from the three types of atmospheres as functions of sliding revolutions, and the film wear life was determined as the friction coefficient exceeded 0.2. Specifically, the lowest friction coefficient was obtained in high vacuum about 0.03 with a wear life of 1.6 × 10^5^ revolutions, whereas higher friction coefficients about 0.07 were seen both in low vacuum and ambient air, nevertheless, with entirely distinct wear lives of 1.6 × 10^5^ and 3.5 × 10^4^ revolutions, respectively. Consistently, in Fig. 6(b) the lowest film wear rate was obtained in high vacuum about 3.8 × 10^−8^ mm^3^/N·m, while a moderate value of 5.1 × 10^−7^ mm^3^/N·m and a higher value of 6.1 × 10^−7^ mm^3^/N·m were seen in low vacuum and ambient air, respectively.

At macroscopic level, these results suggest that the humidity-sensitivity of sputtered MoS_2_ phase is partially restricted by introducing a-C phase into the composite film system. On one hand, the composite film exhibits dense structure and the cracks along the direction of film growth are significantly blocked, so that the humidity-driven oxidation of sputtered MoS_2_ in humid atmospheres (including ambient air and low vacuum in this work) are well restricted^24^,^25^,^30^,^31^,^32^,^33^. On the other hand, the possible formation of active polar bonding between carbon and water molecular in tribochemical reactions may impede the release of non-lubricant phases from tribofilms, so that the low friction in humid atmospheres is achieved with a sacrifice on the large wear of bulk film^20^.

At nanoscopic level, the compositional mixed capping layer with weak lubricant properties in humid atmospheres will be worn out and mechanically released from tribofilms, particularly before the establishment of stable tribofilms, so that the film wear rate in ambient air and low vacuum atmospheres are one magnitude higher than that in high vacuum.

**Figure 7:**
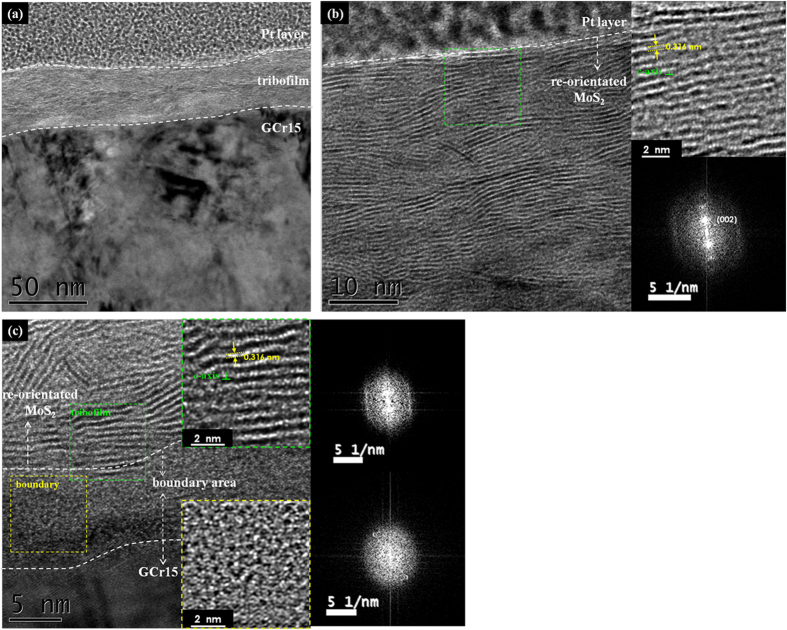
The cross-sectional HRTEM observation of the tribofilms derived from bulk film sliding in high vacuum.

Deeper insights into the lubricant mechanism of bulk film in high vacuum was achieved by cross-sectional HRTEM observations of the transferred materials adhered on GCr15 balls. As shown in Fig. 7(a), a tribofilm in thickness about 30 nm was formed in large parts of the sliding interface, and a closer inspection of its top layer (in Fig. 7(b)) clearly discloses a structural transformation from as-deposited alternating bi-layers to finely-aligned MoS_2_ basal plane in parallel to the shearing direction. The local fast Fourier transformation analysis added on right side of Fig. 7(b) indicates nearly simplex (002) orientation of transferred MoS_2_ phase onto blank counterface. This structure is the key mechanism of steady-state low friction performance of deposited film with long life-time in appropriate atmospheres. It is noted that the thickness of finely-aligned top layer is much thicker than the previously reported few layers of aligned WS_2_ on the top of sliding interface^31^,^34^,^35^,^36^. Compared with the localized clustering structure of MoS_2_ (or WS_2_) nanocrystallites embedded in a-C matrix^31^, the nanoperiod multilayer structure with MoS_2_-riched domain layers in well-aligned (002) orientation, as described in this work, is more favorable for the establishment of low-friction tribofilms and thus results in the lowest friction coefficients and film wear in appropriate sliding atmospheres. In addition, in Fig. 7(c) an amorphous boundary area in thickness about 10 nm is observed between the aligned MoS_2_ basal planes and GCr15 steel surface, while the crystallinity of MoS_2_ phase in green dashed frame is slightly weaker than that of the top surface. This boundary area was formed during the running-in period of sliding from peeled-off asperities on top surfaces of as-deposited films and steel balls, which provided durable adhesion of tribofilm onto blank ball surface.

## Conclusions

In present work, highly ordered MoS_2_/Mo-S-C multilayers with sub-layer thickness in few nanometers were synthesized by a novel self-assembling mechanism in simultaneous sputtering of MoS_2_ and graphite targets. The MoS_2_-riched domain layers preferentially aligned in (002) orientation and Mo-S-C compositional mixed capping layers were altered periodically in a waving pattern that was roughly in parallel to the film surface. The phase segregation was initiated from substrate-film interlayer with clear contrasts in the first tens of nanometer growth and then diffused mutually in a quasiperiodic pattern between two altered sub-layers. Compared with pure sputtered MoS_2_ film, the bulk film of nanoperiod multilayers exhibited largely improved hardness and toughness under a normal load, and the preferential orientation of sputtered MoS_2_ in (002) basal planes was significantly enhanced, both of which rendered the film excellent loads-bearing capacity and lubricant properties. The nano-scratching tests performed on a nanoindentation system revealed that the up and down fluctuation quasi-periodicities of |*F*_*n*_*/F*_*s*_| ratio were roughly consistent to the saturated thickness of compositional mixed capping layers and MoS_2_-riched domain layers, and the nano-tribological performance of multilayers was directly determined by the altered structure and properties of neighboring sub-layers until stable tribofilms were formed on sliding counterparts. Meanwhile, the pin-on-disk tribotests of bulk film in ambient air, low vacuum and high vacuum provided comparably low friction coefficient in the three different atmospheres yet distinct wear lives due to the partially restricted humid-sensitivity of sputtered MoS_2_ phase.

## Methods

### Deposition methods

The MoS_2_/Mo-S-C multilayers were synthesized by applying a multi-target r.f. magnetron sputtering system with a base pressure of 1.3 × 10^−3^ Pa and working pressure of pure Ar gas for 0.75 Pa. The details of the experimental setup are referred to our recent work^20^. Ones of MoS_2_ and graphite targets in diameter of 75 mm and 99.99% purity were simultaneously co-sputtered onto p-type Si(100) wafers with a floating bias voltage, and the vertical distance to the targets was about 80 mm. The r.f. sputtering power of the two targets were the same as 275 W, which resulted in the deposition rates approximately as 30 nm/min for sputtered MoS_2_ phase and 3 nm/min for a-C phase, as determined by a separate study. The sample stage rotated at a constant speed of 1.5 rev./min and was continuously infrared heated to 150°C. The film was deposited for 120 min, causing a film thickness about 2.3 μm.

### Structure characterizations

The morphology and cross-sectional microstructures of deposited films were investigated by using field-emission scanning electron microscopy (FESEM, JSM-6701F, JEOL, Japan) and HRTEM (TECNAI G2 S-TWIN F20, FEI, USA; accelerating voltage 200 kV). The crystallographic phases of deposited films were investigated by using grazing incidence X-ray diffraction (GIXRD, Rigaku RINT2400), and Raman spectroscopy (Horiba LabRam HR800). The GIXRD measurements were carried out using Cu K*α* radiation *λ* = 1.54056 Å and the diffractograms were acquired from 10° to 70°. The Raman spectra were measured by a 532 nm wavelength excitation, using a laser power of D2 filter and one acquisition cycle for 120 s to avoid film damage.

### Mechanical and tribological properties characterizations

The mechanical and nano-tribological properties of deposited films were investigated by using a Nano Indenter DCM nano-mechanical system (MTS, America). The nanoindented hardness and Young’s modulus were determined by using a Berkovich diamond indenter, and the maximum indentation depth was set to be 200 nm (<10% of film thickness) to eliminate the effects of substrate deformation^37^. Instead, the nano-tribological property of deposited films was checked by using a conical diamond indenter with a nominal tip radius of 1.03 μm and vertex angle of 90°. The tip scratched on the film in a gradual-depth-increase mode, and the indentation depth and scratching length were set as 200 nm and 10 μm, respectively. The indentation durations were set as 100 s and 200 s for comparison, which resulted in the indentation speeds of 2 nm/s and 1 nm/s, respectively.

The micro-tribotests were conducted on a standard pin-on-disk tribometer in rotating mode by using ultrasonically cleaned GCr15 steel balls in diameter of 3 mm as the sliding counterparts. The tests were performed under a normal load of 3 N, which created a Hertz contact pressure about 1.5 GPa. The balls were stationary and the disk fixed with tested films rotated in a speed of 1000 rev./min, producing wear tracks in radius of 3 mm on the films. For high vacuum tests, a pressure <3 × 10^−3^ Pa was achieved at room temperature. For low vacuum tests, a pressure of 0.08 Pa was achieved at room temperature, and the remained relative humidity in testing chamber was about 20 ± 5%. For ambient air tests, the relative humidity was about 35 ± 5%. Besides, another set of 2 × 10^4^ revolution tribotests were performed in the three types of atmospheres, and the wear rates of bulk films were evaluated accordingly using the relationship of *K* = *V*/(*LF*_*n*_) where *V* was the wear volume loss in cubic meter, *F*_*n*_ the normal load applied on ball counterpart in Newton and *L* the sliding distance in meter. After friction tests, the transferred materials adhered on steel balls were observed by HRTEM, and the cross-sectional samples were prepared by using focused ion beam (FIB) technique.

## Additional Information

**How to cite this article**: Xu, J. *et al*. Growth and characteristics of self-assembled MoS_2_/Mo-S-C nanoperiod multilayers for enhanced tribological performance. *Sci. Rep*. **6**, 25378; doi: 10.1038/srep25378 (2016).

## Figures and Tables

**Table 1:** The gradient developments of sub-layer thickness from film-substrate interlayer to upward sub-layers.

interlayer (nm)	1		2		3		4		5		6		7		>7	
	1-D	1-B	2-D	2-B	3-D	3-B	4-D	4-B	5-D	5-B	6-D	6-B	7-D	7-B	n-D	n-B
4.6	4.1	2.3	4.6	2.5	5.7	2.7	7.1	2.7	7.3	2.7	8.0	2.1	8.0	2.5	8~9	2~4
